# Perioperative Care in Patients with Chronic Liver Disease: What Can We Do More?

**DOI:** 10.3390/medicina59020384

**Published:** 2023-02-16

**Authors:** Gabriela Droc, Sebastian Isac

**Affiliations:** 1 Department of Anesthesiology and Intensive Care I, Fundeni Clinical Institute, 022328 Bucharest, Romania; 2 Department of Anesthesiology and Intensive Care I, Carol Davila University of Medicine and Pharmacy, 020021 Bucharest, Romania; 3 Department of Physiology, Faculty of Medicine, Carol Davila University of Medicine and Pharmacy, 020021 Bucharest, Romania

Chronic liver diseases represent a prevalent pathology that exerts considerable pressure on healthcare providers and various healthcare systems worldwide. In addition to the vast spectrum of complications that could appear because of disease progression to liver cirrhosis, anesthesia and surgery pose a considerable risk to a patient with chronic liver disease [1]. For a better outcome, major liver surgery in patients with pre-existing liver diseases, regardless of their stages, should be addressed in specialized centers [2].

Special consideration should be focused on perioperative risk assessment strategies and fast-track-adapted surgical protocols. The main goals in preoperative settings are to optimize the fluid balance and coagulopathy, treat hepatic encephalopathy, avoid nephrotoxic drugs, maintain a familiar social environment, and improve nutritional status and cardiopulmonary function. Intraoperative care should focus on goal-directed fluid therapy, a point-of-care analysis of coagulopathy, an assessment of the stress response to surgery due to the appropriate invasive monitoring, sufficient anesthesia depth and nociception assessments, the rational use of various anesthetic drugs, considering the modified pharmacokinetic in cirrhotic patients, and specific surgery-related challenges [3]. 

Postoperative clinical practice should address the following issues: optimal management based on enhanced recovery after surgery (ERAS) protocols; comprehensive pain management; counteracting the postoperative hyperdynamic status; and, regarding hypotension, which is normal in this patient subgroup, identifying and treating postoperative renal impairments; and optimizing neurocognitive status using a multidisciplinary approach [4]. 

Additionally, novel epigenetic biomarkers could serve as potential predictors in identifying the patients at risk for developing various perioperative complications or surgery-related cirrhosis decompensation [5]. Special attention should be focused on recognizing liver-related plasma miRNA (microRNA) and their perioperative dynamics, as well as histone acetylation and DNA methylation of the targeted genes that could influence the postoperative outcome in patients with chronic liver disease. 

Submissions regarding these factors and their possible influence on patient outcomes are welcomed for the current Special Issue entitled “Chronic Liver Diseases and Liver Surgery in Anesthesia” to analyze, prioritize, and identify solutions for better intensive care and surgical practice. 

## References

[1] Rai R., Nagral S., Nagral A. (2012). Surgery in a Patient with Liver Disease. J. Clin. Exp. Hepatol..

[2] Andrei S., Isac S., Carstea M., Martac C., Mihalcea L., Buzatu C., Ionescu D., Georgescu D.E., Droc G. (2022). Isolated liver trauma: A clinical perspective in a non-emergency center for liver surgery. Exp. Ther. Med..

[3] Abbas N., Makker J., Abbas H., Balar B. (2017). Perioperative Care of Patients with Liver Cirrhosis: A Review. Health Serv. Insights.

[4] Zheng Y., Wang L., Wu F., Rong W., Liu Y., Zhang K., Wu J. (2020). Enhanced Recovery after Surgery Strategy for Cirrhosis Patients Undergoing Hepatectomy: Experience in a Single Research Center. Ann. Surg. Treat. Res..

[5] Isac T., Isac S., Rababoc R., Cotorogea M., Iliescu L. (2022). Epigenetics in Inflammatory Liver Diseases: A Clinical Perspective (Review). Exp. Ther. Med..

